# Foods advertised in US weekly supermarket sales circulars over one year: a content analysis

**DOI:** 10.1186/1475-2891-13-95

**Published:** 2014-09-23

**Authors:** Lisa Jahns, Collin R Payne, Leah D Whigham, LuAnn K Johnson, Angela J Scheett, Bonita S Hoverson, Sibylle Kranz

**Affiliations:** United States Department of Agriculture, Agricultural Research Service, Grand Forks Human Nutrition Research Center, Grand Forks, ND 58203 USA; Department of Marketing, New Mexico State University, Las Cruces, NM 88003 USA; Paso del Norte Institute for Healthy Living, 500 W. University Ave, El Paso, TX 79968 USA; Centre for Exercise, Nutrition, and Health Sciences, School of Policy Studies, 8 Priory Road, Bristol, BS8 1TZ USA

**Keywords:** Dietary guidelines, Advertising, Supermarkets, Grocery stores, Promotion

## Abstract

**Background:**

The nutritional content of Americans’ shopping carts is suboptimal despite federal dietary guidance, in this case, the MyPlate consumer icon which displays desired proportions of vegetables, fruits, dairy, grains and protein foods for consumption. Consumers mention print advertising—such as weekly sales circulars—frequently as influencing their grocery shopping decisions.

**Methods:**

To examine and describe the relative proportions of advertised foods aggregated into the MyPlate food grouping system, a content analysis of 9 209 foods advertised in 52 weekly supermarket newspaper sales inserts in 2009 from a local grocery chain was conducted in a Midwestern community.

**Results:**

Overall, the protein foods group was most often represented in sales circulars (25% of total items), followed by grains (18%); dairy (10%); vegetables (8%) and fruits (7%). Less than 3% of sales advertisements were for dark green and red & orange vegetables. Over twice as much whole fruit versus 100% fruit juice was advertised (70% vs. 30%, respectively; *P* < 0.001). Significantly fewer protein foods and more grains than expected were advertised in the fall, and slightly more dark green vegetables were advertised in winter and spring than in summer and fall (*P* = 0.05).

**Conclusions:**

The average American diet, including underconsumption of fruits and vegetables but overconsumption of protein foods, was reflected in the relative frequency of food groups advertised in weekly sales circulars. Modifying sales circulars to represent healthier food groups may preserve retail profits (considering these groups’ higher profit margin) while promoting adherence to federal dietary guidance.

## Background

Americans reported spending an average of ~ $400.00 USD each month at supermarkets in 2012, and spending has increased slightly since 2006 [1]. While some of the monthly expenditure can be explained by consumer demand for specific food items (e.g., organic products), more can be explained by supermarket strategies to promote specific products that maximize store profitability [2, 3]. One very effective way supermarkets promote specific products is through weekly sales circulars, both in print and online.

Weekly sales circulars provide information to consumers about not only price discounts, but also what foods to consider purchasing. Price discount information is important considering economic downturns and rising food prices [1, 4–8]. Indeed, 88% of consumers say that price is somewhat or very important when buying food [9]. Information regarding what foods to purchase is important considering consumers have to contend with multiple possible grocery stores in which to shop and an average of 39 000 items from which to choose within each store [10]. Weekly grocery store sales circulars help facilitate food purchasing decisions [11].

Over 70% percent of US adults read newspaper circulars [11, 12]. Half of shoppers report using technology when grocery shopping and 23% of these shoppers report that they check prices at multiple stores before shopping [1]. Circulars have been shown to increase targeted versus untargeted item purchasing by 100% [13]. In fact, supermarket sales circulars are so effective in stimulating demand [13–16] that it is difficult to find a supermarket that does not use them.

Considering weekly sales circulars’ effectiveness on influencing consumer purchases, it is not surprising that seasonal changes in promotion would affect consumer purchase types and amounts. For example, there is evidence that food pricing and advertising vary seasonally [17], as do purchases of vegetables and fruits [18]. Furthermore, seasonality may be associated with food intake [19–21], suggesting that advertising different foods more heavily at certain times of the year, such as vegetables and fruits in the summer or meats over holidays, may influence demand. Despite being an important new area of research given the influence of advertising on purchasing and its potential to positively impact dietary habits, the healthfulness of food items advertised in sales circulars is understudied. To our knowledge, there have been only two short-term studies published to date [22, 23] that have described the contents of weekly grocery store sales circulars. Examination of supermarket sales circular content is critical to diet quality research to help understand commercial promotion of food types, which—inadvertently or advertently—are used as consumption recommendations [24]. Given that American’s food purchases [25] and dietary intake [26] fall short of the Dietary Guidelines for Americans (DGA) [27] recommendations, and that supermarket sales circulars are widely used by consumers to guide their purchases, circulars have the potential to have a positive influence on an individual’s diet quality [24].

Accordingly, the goal of this study was to examine the content of a year’s worth of sales circulars to determine whether food groups advertised in sales circulars varied from recommended food group intake guidance overall and by season. We hypothesized that circulars would reflect seasonal changes and advertise more vegetables and fruits in summer and fall compared to the winter and spring seasons.

## Methods

Fifty-two weekly supermarket sales circulars dating from January 1 to December 31, 2009 were collected from a local Midwestern supermarket chain. The chain consists of eight stores located in a city of 69 000 predominantly Non-Hispanic white individuals (86.7%) [28], and is considered small compared to other chain supermarkets [29]. As all stores belonged to the same chain, there was no variation in the circulars by individual store. Each food item in the weekly circulars was dual-coded by trained research personnel to assure data entry accuracy; all discrepancies were resolved by a supervisory research dietitian. A total of 9 209 food items were coded. The coding scheme for the advertised items was as follows: all items advertised in the circulars were classified as food or nonfood items. Food items were further classified into food groups and subgroups of food groups. Guidelines for classifying were based upon the following: The initial groupings were based on the Food Group Description File from the USDA National Nutrient Database for Standard Reference, Release 22 [30] and subgroups from USDA Handbook 8 (AH8) [31] with minor modifications to include products not originally designated in AH8. Food items were then grouped by major food groups (vegetables, fruits, grains, protein foods, and dairy) and subgroups of MyPlate, the consumer icon implementing the DGA [32] (n = 6 366; 69%) using the MyPyramid Equivalents Database [33]. The DGA makes recommendations to limit or reduce intake of the food components termed empty calories (solid fats and added sugars), therefore this group was also included. The solid fats group contains fats that are solid at room temperature, such as margarine spreads and added sugars refers to sugar that is not naturally-occurring and is added to foods during processing. The added sugars group in this study includes foods that are comprised predominantly of added sugar, such as sugar-sweetened beverages and hard candy [32]. Advertised items that did not have enough detail to determine the food group were categorized as “other” (n = 2 843). The “other” group included mixed dishes that could not be categorized into food groups. Wherever possible, the major food groups were disaggregated into subgroups. For instance, the fruit group was divided into whole fruit and 100% fruit juice, and dairy, protein, and vegetable groups were broken into individual components. It was not possible to identify whole versus non-whole grain products from the advertisements, however, we subjectively categorized grain products as “with” or “without” added sugars. For instance, cakes, cookies, and sweet rolls were categorized as “with added sugar” while bread, pasta, and crackers were classified “without added sugar”. Seasons were categorized using the meteorological definition as follows: winter (December, January, February), spring (March, April, May), summer (June, July, August), and fall (September, October, November).

To determine whether observed frequencies of the five recommended food groups in the advertisements (n = 6 366) varied significantly from what was expected across seasons, a contingency table analysis was conducted using PASW Statistics 18, Release Version 18.0 [34]. Significant discrepancies between observed versus expected frequencies were determined by computing adjusted chi-square residuals for each contingency table cell. As adjusted chi-square residuals are normally distributed, any residual value greater than or equal to ±1.96 is interpreted as a significant departure from what was expected [35].

A generalized linear model was used to test for seasonal differences in the relative proportions of advertised items for each food group and subgroup using Proc GENMOD in SAS Version 9.2 [36]. If the overall model was significant, Tukey’s contrasts were used to do pairwise comparisons between all seasons.

## Results

**Figure 1:**
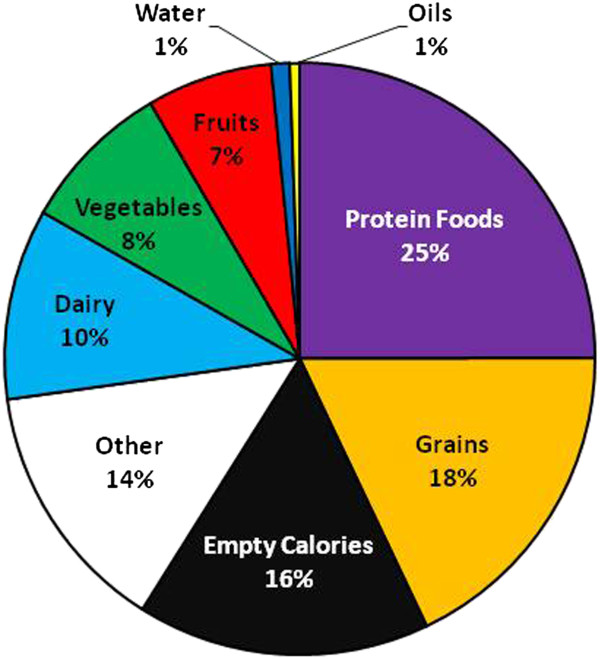
**Proportions of food groups advertised in 52 weekly US supermarket sales circulars in 2009.**

**Figure 2:**
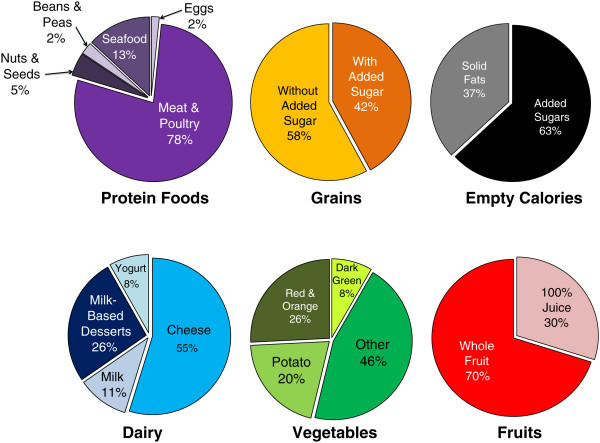
**Proportions of food subgroups advertised in 52 weekly US supermarket sales circulars in 2009.**

The proportions of all food and subgroups advertised over the year are presented in Figures 1 and 2. Over the year, the groups frequently advertised of the recommended food groups was the protein foods group, which comprised one-fourth of all items described. Grains were the next most often advertised group (18%), followed by dairy (10%), vegetables (8%), and fruits (7%). Sixteen percent of foods were categorized as empty calories while another 14% were not able to be categorized. Meat, poultry and cheese were the most often advertised items in the protein and dairy groups, respectively.

**Figure 3:**
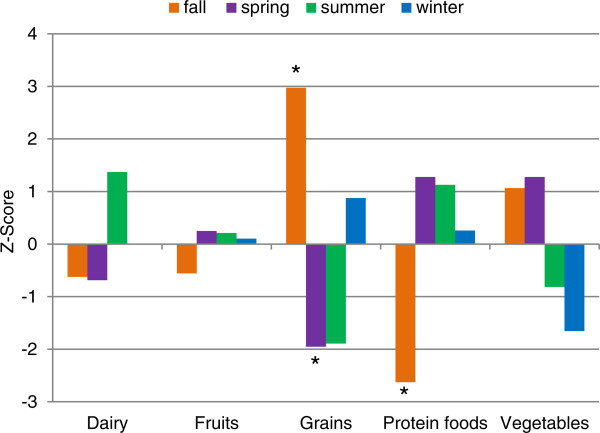
**Expected compared to observed frequency of food groups advertised seasonally.** Footnotes: Adjusted chi-square residuals were computed for each contingency table cell. Any residual value greater than or equal to ±1.96 is interpreted as a significant departure from what was expected in each season and marked with an *.

Sixty-nine percent of all food items were categorized into the five major food groups. The frequency of items advertised approached significance as a function of season (overall chi-square test of independence, *χ*^2^ (12, *N* = 6, 316) =20.1, *P* = 0.066). The adjusted chi-square residuals are plotted in Figure 3. Advertised frequencies of grains were significantly higher in fall (*z* = 2.97, *P* = 0.003) and lower than expected in the spring (*z* = 1.95, *P* = 0.051). The frequency of protein foods advertised was lower than expected in fall (*z* = 2.62, *P* < 0.009). There were no significant differences in the observed frequencies in the vegetable, fruit, or dairy groups by season.

Next, we examined differences in groups and subgroups by season using generalized linear regression (Table 1). Items in the grain group were advertised significantly more often in fall compared to spring or summer (*P* < 0.001) and grains without added sugar was lowest in the spring months (*P* < 0.001). There were no seasonal differences in frequency of advertisments of grains with added sugar. Overall, there were no significant differences in the dairy group (*P* = 0.35), but fluid milk advertisements varied seasonally (*P* = 0.02). Advertised dark green vegetables also varied by season (*P* = 0.05); as did seafood, which was more heavily advertised in the winter months (*P* < 0.001).

**Table 1:** **Differences in the proportions of food groups advertised in weekly supermarket sales circulars by season** ^**1**^

	Season								
	Winter		Spring		Summer		Fall		
	n = 2 006		n = 2 622		n = 2 154		n = 2 427		
MyPlate major food groups and subgroups	n	%	n	%	n	%	n	%	p-value
Grains	388	19.3^ac^	427	16.3^b^	360	16.7^ab^	481	19.8^c^	< 0.001
Without added sugar	224	11.2^ac^	233	8.9^b^	205	9.5^ab^	298	12.2^c^	<0.001
With added sugar	164	8.2	194	7.4	155	7.2	183	7.5	0.67
Vegetables	156	7.8	227	8.7	171	7.9	214	8.8	0.50
Other vegetables	65	3.2	114	4.4	85	4.0	85	3.5	0.20
Red & orange	40	2.0	47	1.8	48	2.2	63	2.6	0.25
Potatoes	34	1.7	40	1.5	29	1.4	54	2.2	0.12
Dark green	17	0.9	26	1.0	9	0.4	12	0.5	0.05
Fruits	144	7.2	177	6.8	150	7.0	160	6.6	0.88
Fruit	94	4.7	126	4.8	115	5.3	107	4.4	0.53
100% fruit juice	50	2.5	51	2.0	35	1.6	53	2.2	0.23
Oils	9	0.5	13	0.5	7	0.3	21	0.9	0.08
Dairy	218	10.9	257	9.8	242	11.2	245	10.1	0.35
Cheese	125	6.2	143	5.5	134	6.2	125	5.2	0.29
Milk-based desserts	44	2.2	76	2.9	72	3.3	62	2.6	0.13
Fluid milk	31	1.6	21	0.8	16	0.7	34	1.4	0.02
Yogurt	18	0.9	17	0.7	20	0.9	24	1.0	0.55
Protein Foods	525	26.2	657	25.1	557	25.9	560	23.1	0.66
Meat & poultry	391	19.5^ab^	513	19.6^ab^	457	21.2^a^	433	17.8^b^	0.04
Seafood	97	4.8	85	3.2^a^	50	2.3^a^	70	2.9^a^	< 0.001
Nuts and seeds	20	1.0	30	1.1	23	1.1	36	1.5	0.45
Beans & peas	10	0.5	18	0.7	16	0.7	13	0.5	0.69
Eggs	7	0.4	11	0.4	11	0.5	8	0.3	0.78
Water	21	1.0	30	1.1	20	0.9	23	1.0	0.87
Empty calories	300	15.0	408	15.6	376	17.5	388	16.0	0.15
Added sugars	182	9.1	253	9.7	242	11.2	252	10.4	0.10
Solid fats	118	5.9	155	5.9	134	6.2	136	5.6	0.85
Other^2^	245	12.2^a^	426	16.3^b^	271	12.6^a^	335	13.8^ab^	< 0.001

^1^Values with the same superscript letters are not significantly different from each other.

^2^Includes combination foods; not enough detail available to separate out into groups.

## Discussion

In this study we described the seasonal content of one year’s worth of Sunday supermarket circulars. There was little variation between the frequencies of food groups advertised by season. In particular, advertisements for the major fruit and vegetable groups did not vary significantly during the summer or fall seasons, as one would expect, since one might assume that they are more easily available to supermarkets during the harvest months of the year. Only the promotion of dark green vegetables varied by season, with the highest numbers in spring compared to summer, although the absolute number of items advertised was neligible. However, advertisements for grains were greater in the fall and lower in spring whilst the frequency of protein foods was lower in the fall season than expected. The increased advertising of grain products in the fall was not due to changes in the grains with added sugar category, as might be anticipated with the demand for holiday baked goods, such as cookies and other sweetened desserts, but due to changes in the grains without added sugar category.

Overall, the frequency of advertisements for most food groups by this supermarket chain did not reflect current recommendations. Although nearly 50% of the recommended foods by the DGA are fruits and vegetables, we found that only a small proportion of advertisements were for items from those two food groups (15%). Encouragingly, one-fifth of items advertised were grain products. Although we were unable to distinguish whole grain items from refined items, we roughly categorized grains with added sugar and grains without added sugar, and found that over half of all grain products advertised were in the without added sugar category. As many food manufacturers are reformulating their grain products to contain more whole grains, the relatively high frequency of advertisements for grain products without added sugar has the potential to increase whole grain purchases and consumption [37].

Our findings are similar to those of Martin-Biggers et al. [22], who to our knowledge were the first investigators to compare the content of supermarket flyers to dietary recommendations and obesity prevalence in a national sample of sales circulars. Their approach compared the physical space devoted to the major food groups recommended by the DGA on the front page of single weekly supermarket sales circulars nationwide to the space allocated on the MyPlate icon. Despite the fact that their approach differed significantly from ours and they did not examine advertisements over time, they also found that while protein foods were over-represented, dairy, fruits, and vegetables were underrepresented; only the grains group was represented in approximately the same proportions as those shown in the MyPlate icon. It is of interest that the same pattern was found both when examining either the front page only or the entire circular, and either in a national sample at a single time point or in a regional sample over a year. Ethan et al. reported the nutrition content of items on the first page of online sales circulars in the Bronx area [23]. They found that, at least on the front page, approximately 16% of advertisements were for vegetables or fruits, including 100% juice, compared to our findings of 15% and also reported that grain products accounted for 15% of ads compared to our results of 18%. Again, using different methods and time periods, results are consistent, indicating that perhaps overall, the front page of circulars is representative of the content of the full advertisement. Regardless of the methodology, this important initial research consistently points to a lack of concordance between dietary guidance and food items advertised in sales circulars.

Compared to recommendations, purchases for fruits and vegetables are suboptimal while products high in solid fats and added sugars are excessive [25]. While there is little research linking food purchases to food consumption [38], a growing body of literature suggests that price reductions or provision of coupons and food vouchers can improve both purchases and dietary intake of healthy food [39]. Online coupons are becoming popular with retailers; however, current research indicates that online coupons are overwhelmingly for processed snack foods, not for healthier foods [40]. Many intervention efforts to promote the consumption of nutrient dense foods, especially fruits and vegetables, have turned to retail outlets. Economic incentives are often coupled with behavioral change strategies such as price manipulation, targeted coupons, in-store education, printed promotional materials, changes in product placement, and newspaper messages [41]. Other novel approaches include influencing purchasing by manipulating social meaning during shopping [42] and providing direct guidance for fruit and vegetable recommendations built into shopping carts [43]. Yet, to our knowledge, none have partnered with stores to target weekly circulars as a method of promoting price discounts of healthy, nutrient dense, foods. Relatively minor and inexpensive changes in content and placement of advertised items in circulars hold great potential to influence purchasing behavior and dietary intake to more closely align with recommendations.

The sales circulars were from a local grocery chain in the Midwest and may not reflect advertised products in other parts of the country. Other locations may also have a more pronounced seasonal variation in items advertised, especially vegetables and fruits, however, as our results were not dissimilar to other studies [22, 23], they may be relevant to other parts of the country. Although coders worked in teams, coding was subjective and not all advertisements could be accurately classified, such as promotions where a variety of food items by one company were advertised together. We could not discriminate refined grains from whole grains as generally advertisements for ready-to-eat cereals and breads contained a variety of both whole- and non-whole grains. Because we counted each advertised item as it was displayed, we did not account for “buy one get one free” multiples. Although we did not distinguish between high- and low-fat meats or fried vs fresh fish, food items were categorized by sub-categories of the major MyPlate food groups. Federal guidance recommends decreasing intake of solid fats and added sugars, but this is not reflected in the MyPlate icon. Strengths of the study include the use of foods in the entire circular, the use of a full years’ worth of advertisements, and the comparison to federal dietary guidance. This novel approach adds to the sparse literature on grocery store sales circulars.

Stores use sales circulars to increase purchases by existing customers and to attract customers away from their usual grocery store by offering price discounts. However, if the sales circulars are not only interpreted as communicating purchasing deals but also function as intake guidance, then the relationship between sales circulars, purchasing behavior, and food consumption patterns needs to be investigated further.

Using a calendar year of supermarket sales circulars to ascertain information on the types of foods advertised helps us to understand consumer choices and can inform policy changes to promote healthier diets. Consumers indicate that weekly sales circulars are main factors in food purchasing decisions; thus, modification of the items advertised has the potential to significantly support people’s efforts to eat healthier.

Reconfiguring sales circulars to promote healthier items may result in at least four possible outcomes: 1. No effect (i.e., new circulars are ignored); 2. A decrease in total sales and sales of healthier foods (i.e., consumer reactance to new circulars); 3. A decrease in total sales, but increase in healthier food sales (i.e., switching from less healthy food to more healthy food because of new circulars); 4. An increase in total sales and sales of healthier foods (i.e., keeping current purchasing patterns plus new healthy circular foods). Given what is known about price discounts and in-store marketing [24, 43], we would expect outcomes #3 or #4. Currently, however, there is no empirical evidence examining the impact of systematic circular changes on purchases of healthier foods. Consequently, we expect this to be a fruitful area of future research regarding attempts to encourage healthier purchases. Increased focus on promoting fruits and vegetables in sales circulars may result in increased retail profits, through promotion of sales and simultaneous reduction in waste due to spoilage [44, 45] —a beneficial effect for the stores that would likely keep these foods consistently in weekly circulars.

## Conclusions

The results of this study demonstrated that the food items advertised in weekly grocery circulars in this particular geographic location did not correspond to the current DGA recommendations. Nevertheless, health interventions via supermarket advertising are largely underexplored to date. If changes in advertisements were partnered with interventions sensitive to the needs of retail stakeholders, the goal to increase the proportion of Americans meeting national dietary guidance, thereby decreasing the risk of chronic disease, could be paralleled by increased profits for the participating retailers.
